# Infrared Near-Field Spectroscopy of AlGaN/GaN Heterostructures for Probing Two-Dimensional Electron Gas

**DOI:** 10.1021/acsami.5c12417

**Published:** 2025-08-22

**Authors:** Ilario Bisignano, Masataka Imura, Nicholaus Kevin Tanjaya, Ming-Jyun Ye, Noriyuki Okada, Satoshi Ishii

**Affiliations:** † International Center for Materials Nanoarchitectonics (MANA), 52747 National Institute for Materials Science (NIMS), Tsukuba, Ibaraki 305-0044, Japan; ‡ Graduate School of Science and Technology, University of Tsukuba, Tsukuba, Ibaraki 305-8577, Japan; § Research Center for Functional Materials, National Institute for Material Science (NIMS), Tsukuba, Ibaraki 305-0047, Japan; ∥ College of Photonics, National Yang Ming Chiao Tung University, Tainan 711010, Taiwan; ⊥ Research Network and Facility Services Division, National Institute for Materials Science (NIMS), Tsukuba, Ibaraki 305-0047, Japan

**Keywords:** near-field microscopy, infrared spectroscopy, AlGaN/GaN heterostructure, two-dimensional electron gas, nano-FTIR

## Abstract

Infrared near-field spectroscopy, or nano-FTIR, offers nanoscale resolution in three dimensions to probe the chemical and physical properties of samples, making it a unique characterization tool. This nanoscopic resolution in three dimensions is particularly suitable to probe a two-dimensional electron gas (2DEG) where a 2DEG has an effective thickness of a few nanometers and exists a few tens of nanometers below the capping layer. This work employs nano-FTIR spectroscopy to noninvasively probe the 2DEG of AlGaN/GaN heterostructures, which are crucial for high-power electronic devices and sensing applications. Higher harmonic amplitude and phase of the nano-FTIR spectra are sensitive enough to the carrier concentration of the 2DEGs, which is supported by analytical calculations based on the finite dipole model. A comparative analysis confirms that incorporating the 2DEG layer into the model is essential to matching spectral features with experimental observations. Furthermore, hyperspectral imaging of a cross-sectional sample provides a visual representation of the 2DEG. The findings demonstrate that nano-FTIR enables the characterization of 2DEG in AlGaN/GaN heterostructures with nanometric resolution under ambient conditions, hence expanding its applicability in the study of such systems.

## Graphical abstract

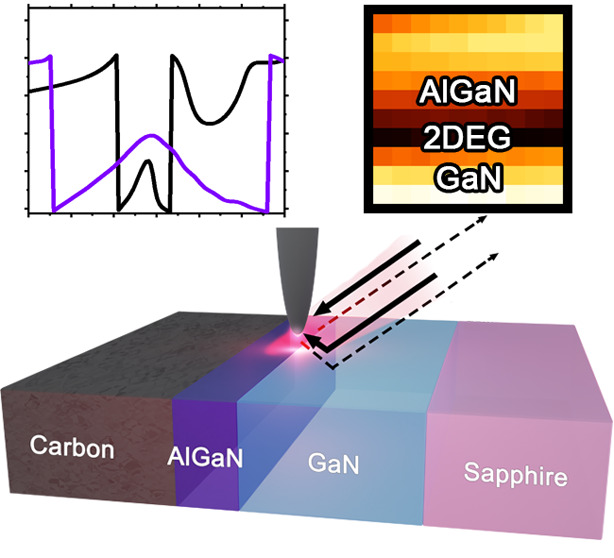

## Introduction

AlGaN/GaN heterostructures have garnered significant attention for their exceptional electromagnetic properties over a wide frequency range,^1,2^ making them highly relevant for various applications including sensing^3–6^ and THz detection.^7,8^ In electronics, power devices find a wide use of GaN heterostructures^9^ but are affected by temperature degradation, such as in AlGaN/GaN FinFETs,^10^ which makes exploring their heat transfer and emission properties an important point to be considered for future applications and characterizations. The plethora of applications of these heterostructures requires an ever-increasing need for accurate and detailed characterization of the structure’s properties, especially at the nanoscale, with the increasing trend of downscale devices.^11^ This crucial need underlines the importance and motivation of the present study.

Of the commonly used characterization processes, the electrical transport in 2DEG materials is measured via Hall effect measurements,^12^ magnetoresistance measurements,^13^ split C–V measurements,^14^ Kelvin probe,^15^ or by exploiting the oscillations in the conductivity at low temperatures and high magnetic fields in the Shubnikov–de Haas effect.^16,17^ Optical characterization of the 2DEG is usually carried out by photoluminescence experiments;^18,19^ however, identifying the 2DEG’s most prominent feature requires the disappearance of a peak after etching the AlGaN layer, making it a destructive technique. Moreover, the characterization of the electron energy and momentum, which unveils information about dispersion relations, is carried on via angle-resolved photoemission spectroscopy.^20^ Regarding the visualization of 2DEG, the only available techniques are differential phase contrast scanning transmission electron microscopy^21^ or scanning tunneling microscopy.^22^

Nano-FTIR offers a different approach to characterizing 2DEG samples; it combines scattering-type scanning near-field optical microscopy (s-SNOM) with Fourier transform infrared (FTIR) detection. In nano-FTIR, a broad-band infrared (IR) laser is focused on the probe apex, generating a highly enhanced near-field between the tip apex and the sample. The backscattered radiation enables the derivation of the sample properties by analyzing its spectroscopic amplitude and phase.^23^ Nano-FTIR has the merit of noninvasive characterization of samples at the nanoscale in ambient conditions.^24–26^

The nanoscale resolution of nano-FTIR is not limited to the lateral direction. Because of the near-field interaction between the probe and sample, the measured spectra contain information from a definite depth within the sample. This probing depth depends, among other parameters, on the demodulation harmonics: as the demodulation order increases, the probing depth decreases, limiting the probing depth to a few tens of nanometers from the uppermost surface. This pivotal point makes nano-FTIR a valid technique to characterize multilayer samples and recover material properties.^27–29^

Because of that, nano-FTIR has been used to investigate the 2DEG in the past; reports mostly focus on oxide/interface 2DEGs (LaAlO_3_/SrTiO_3_ and related)^30–32^ where particular focus was given on identifying the carrier concentration and mobility, while THz s-SNOM has been used for plasmonic characterization of graphene FET channels at nanometer resolution.^33^ To the best of our knowledge, this work presents the first demonstration of nano-FTIR spectroscopy applied to detect 2DEG in AlGaN/GaN heterostructures. Furthermore, it is the first to prove a spatially resolved visualization of 2DEG through hyperspectral imaging on a cross-sectional sample.

This work focuses on probing the 2DEG of AlGaN/GaN heterostructures through nano-FTIR measurements and comparing the measured spectra with analytical calculations. The analytical method using finite dipole methods (FDMs) demonstrated that the 2DEG inclusion was necessary for reproducing the nano-FTIR spectra. While the limitations and approximations inherent in the FDM model were acknowledged, this study achieves notable agreement between the simulation and experimental data. The FDM model contrasts with the finite element method, which is heavily limited by computational constraints.^34^

## Methods

The 2DEG samples were fabricated by initially growing 2.8 μm of GaN buffers on sapphire substrates and subsequentially AlGaN layers via a metal–organic chemical vapor deposition system (SR2328HT-RR, TAIYO NIPPON SANSO CORPORATION). Three samples with AlGaN thicknesses of 15, 30, and 45 nm were prepared and labeled with their AlGaN thickness. In addition, to obtain a cross section of the 45 nm thick AlGaN sample (Figure 1b), a carbon layer was deposited on the AlGaN layer inside the focused ion beam (FIB) instrument (Ethos NX5000, Hitachi High-Tech Corporation) to extend the surface area of the cross section before the cross-sectional fabrication. The cross section was obtained via FIB using argon (Ar) milling at a tilt angle of 10°. The Ar milling was performed by rotating the sample stage four times with 30 s of irradiation at each position and repeated twice; thus, the total Ar milling time was 240 s.

**Figure 1:**
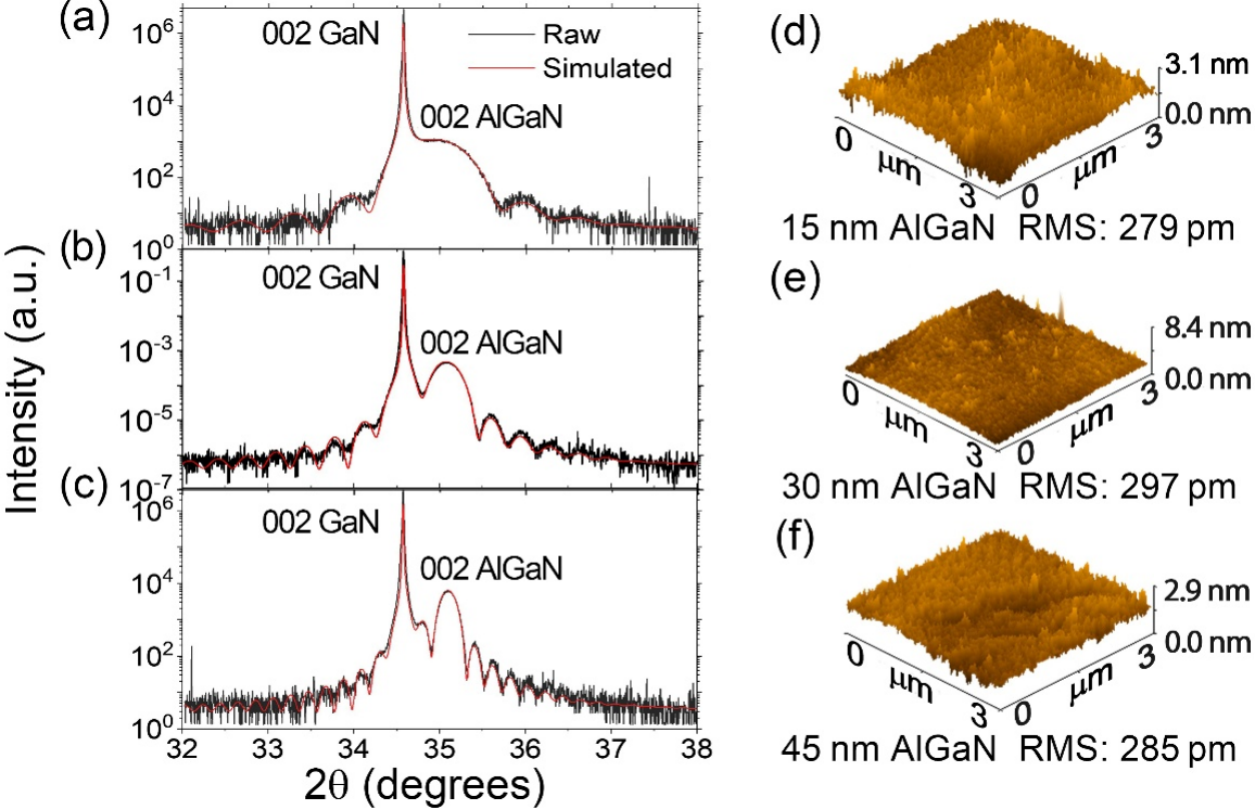
(a–c) Measured (in black) and fitted (in red) XRD patterns and (d–f) AFM images of the (a, d) 15 nm AlGaN, (b, e) 30 nm AlGaN, and (c, f) 45 nm AlGaN samples where AFM scanned the AlGaN surfaces.

The X-ray diffraction (XRD) patterns were obtained by a Smartlab (Rigaku Holdings Corporation). The fitting was performed to determine the thickness and Al content of the AlGaN layer, which were incorporated into the FDM model alongside the average sheet resistance. At the fitting, the in-plane lattice constant of the AlGaN layer was assumed to match that of GaN due to the coherent AlGaN growth. Electron density and band edges were calculated using Nextnano, a technology computer-aided design software used to model and analyze nanoscale semiconductor structures and devices.

To characterize the 2DEG of the samples, Nextnano, a commercial software, was used. Nextnano basically solves self-consistent solutions for the Schrödinger–Poisson equation. The used electronic band parameters are taken from Vurgaftman et al.,^35^ while Nickel Schottky barriers are used as the boundary conditions.

Nano-FTIR measurements were conducted using a commercial s-SNOM (neaSCOPE, Attocube Systems AG) with a broad-band infrared laser covering 8–16 μm. An AFM tip (NSG30Pt, TipsNano) was operated in tapping mode with an initial tapping amplitude of 90 nm and a tip resonance frequency of 297 kHz for background suppression. FTIR-based detection was used to determine the amplitude (*s*_*n*_) and phase (φ_*n*_) of the complex scattering coefficient σ = *s*_*n*_ *e* ^*i*φ_*n*_^ at the second demodulation order (*n* = 2). All of the collected spectra were normalized against a silicon substrate commonly used for calibration (TGQ1, TipsNano) due to silicon’s high reflectance in the mid-IR range. The top-view sample amplitude spectra were collected at three different positions and then averaged. The cross-sectional sample was analyzed by hyperspectral imaging with a scan area of 61 nm × 138 nm with 10 pixels in both directions. The interferometer center and distance were set to 400 and 800 μm, respectively. Each spectrum was averaged 6 times with an integration time of 50 ms.

The reflection was measured using an integrated sphere (Mid-IR IntegratIR, PIKE Technologies, Inc.) combined with an FTIR spectrometer (Nicolet iS50, Thermo Scientific).

The sheet resistance was measured with a noncontact sheet resistance measurement instrument (LEI-1510, Semilab Inc.).

## Results and Discussion

Figure 1 shows the XRD patterns and AFM images of the three AlGaN/GaN samples with variable AlGaN thicknesses of 15, 30, and 45 nm. In the following, the three samples are labeled, depending on the AlGaN thickness. The fitting of the XRD patterns determined the thickness and the Al contents, which were 0.286 for all of the samples. The AFM images show that all of the samples are smooth, having an RMS smaller than 1 nm. A commercial software, Nextnano, was used to simulate the 2DEG layers. The simulations in Figure 2a show that the carrier concentration increases with increasing AlGaN thickness. This thickness dependence aligns with the measured sheet resistance, which decreases with increasing AlGaN thickness. The carrier concentration is dependent on the AlGaN thickness:^36^ as the thickness increases, the triangular potential becomes deeper, resulting in a more confined 2DEG. In other words, increasing the film thickness allows a higher number of free electrons to accumulate at the interface. When this thickness is increased beyond a critical value, however, lattice relaxation occurs, decreasing the polarization charge and eventually leading to a decrease in the carrier concentration. On the other hand, when the AlGaN thickness is decreased from the optimum value, even though the polarization charge is present, the potential is too shallow for sufficient confining, leading to a decrease in carrier concentration, and in the case of extremely thin layers, there is a risk of increased leakage due to tunneling effects.

**Figure 2:**
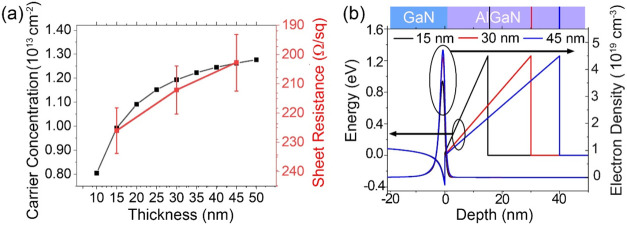
(a) Calculated carrier concentration (in black) and measured sheet resistance (in red) plotted against AlGaN layer thicknesses. (b) Calculated band edges and electron density by Nextnano. In the *x*-axis, the GaN and AlGaN layers are located in negative and positive regions, respectively.

Figure 2b shows the simulated energy level and electron density, which show the existence of the 2DEGs in the GaN sides of the AlGaN/GaN interfaces. Even though the electron density increases with increasing AlGaN thickness, the effective 2DEG width remains nearly identical.

The second-order optical amplitude (O2A) spectra were measured for the three AlGaN/GaN samples and the GaN sample and compared to the modeled O2A utilizing a Python package for modeling scanning near-field optical microscopy measurements.^37^ The fundamental equation governing the SNOM scattering, eq 1, defines the scattering coefficient as

1 $$\sigma =\frac{E_{\mathrm{scat}}}{E_{\mathrm{inc}}}={\left(1+c_{r}r\right)}^{2}\alpha_{\mathrm{eff}}$$

This equation contains information regarding both the near-field interaction (α_eff_) and the far-field radiation reflected from the substrate onto the tip (1 + *c*_r_ *r*).

To compute the scattering coefficient, the FDM was implemented in the multilayer mode, which resulted in a more accurate description of the experimental data compared to the Q average model, as shown in the second-order harmonics data in Figure S1. The second-order harmonics response is used for background suppression, which is considered in the model by simulating lock-in amplifier demodulation at the second harmonics.^37^ The O2A multilayer model better reproduces the features found in the measured data, particularly from 600 to 700 cm^–1^ of GaN phonon resonances, while for the second-order optical phase (O2P), it reproduces the same features at 720 cm^–1^, giving comparable results. Completely opposite is the simulated result from the charge average model, which fails to reproduce any of the previously mentioned distinctive attributes in both O2A and O2P.

By dividing the scattering coefficient of the modeled sample with the scattering coefficient of a reference sample (in our case, silicon), the near-field contrast is obtained and expressed as $\eta_{n}=\frac{\sigma_{n}}{{\sigma_{n}}^{\mathrm{Si}}}=s_{n}e^{i\varphi_{n}}$ . All of the presented simulation results plot O2A and O2P of the complex-valued near-field contrast as a function of wavenumber. Prior to that, the sample multilayer characteristic was taken into account by defining a multilayered substrate with the multilayer method proposed by Hauer et al.^38^ An essential parameter of the model is the AFM tip curvature radius *r*_*tip*_ = 10 nm, which was obtained by comparing experimentally obtained approach curves on silicon for demodulation orders *n* = 2 and *n* = 3 with the simulated ones of varying tip radii, shown in Figure S2a–c. In Figure S2, the normalized second and third harmonic scattering amplitudes of a silicon substrate for different tip height values are compared to the simulated tip effective polarizability demodulated at the second and third harmonics for the same tip heights. The comparison shows that the optimized tip radius is 10 nm, and larger (Figure S2a–c) and smaller values (Figure S2d) lead to curves that deviate far from the experimental ones. The approach curve on silicon is commonly used to validate simulation parameters, especially geometrical parameters, by comparing analytical results with measured ones.^39^ Further confirmation is found when calculating the optical response of the 45 nm thick sample at different tip radii, as shown in Figure S2e,f. When the tip radius is too large, a red shift in the O2A dip caused by the GaN phonon resonance is observed (Figure S2e), which contrasts with the measured spectrum. Also, in the O2P spectrum, at higher tip radii, a second peak appears at 735 cm^–1^ (Figure S2f), which contrasts with the experimentally observed data (Figure S2f in violet solid line).

Other crucial parameters include the finite dipole length *L* = 300 nm, tapping amplitude *A*_tip_ = 72 nm obtained from the experimental conditions, light incidence angle θ_in_ = 60°, and the weighting factor *c*_r_ = 0.9. The top-view sample was modeled as a semi-infinite substrate of GaN with permittivity (eq 2) described as an isotropic single Lorentzian oscillator

2 $$\varepsilon_{\mathrm{GaN}}=\varepsilon_{\infty ,\mathrm{GaN}}\left(1+\frac{\omega_{\mathrm{LO},\mathrm{GaN}}^{2}-\omega_{\mathrm{TO},\mathrm{GaN}}^{2}}{\omega_{\mathrm{TO},\mathrm{GaN}}^{2}-\omega^{2}-i\omega \gamma_{\mathrm{GaN}}}\right)$$

where the wurtzite GaN phonon frequencies utilized were taken from the experimental literature,^40^ which describes the AlN phonon red shift well due to the lattice mismatch.^41^ On top of the GaN semi-infinite layer, a 5 nm thick virtual 2DEG layer was modeled (eq 3) with permittivity^42^

3 $$\epsilon_{2\mathrm{DEG}}=1+i\frac{\sigma_{2\mathrm{DEG}}\left(\omega \right)}{\omega d}$$

The 2DEG layer was capped with an AlGaN layer, where the permittivity was described as a combination of the GaN and AlN permittivities as ϵ_AlGaN_ = xϵ_AlN_ + (1 – *x*)­ϵ_GaN_ with concentration *x* = 28.6% obtained from the XRD measurements. Lastly, the multilayer model ended with a top semi-infinite air layer described by constant permittivity ϵ_Air_ = 1. In detail, the surface conductivity of the 2DEG layer was computed using eq 4 in the Drude model approximation

4 $$\sigma_{2\mathrm{DEG}}\left(\omega \right)=\frac{e^{2}N_{s}\tau }{m^{*}}\frac{1}{1-i\omega \tau }$$

where *N*_s_ is the carrier concentration, *e* is the electron charge, m* is the effective electron mass, and τ is the scattering time defined as $\tau =\frac{m^{*}\mu }{e}$ , with μ being the electron mobility, all of which were obtained using the typical experimental data found in the literature.^43,44^ The carrier concentration *N*_s_ is dependent on the AlGaN thickness and was derived for each sample by utilizing the measured average sheet resistance *R*_sh_ as an input in eq 5 ^45^

5 $$\mu =\frac{1}{eN_{s}R_{\mathrm{sh}}}$$

While for the AlN permittivity, an identical single Lorentzian oscillator model used for the GaN layer was adopted, where the strained AlN phonon frequencies were taken from the literature.^40^ For the silicon reference model, a semi-infinite layer of silicon with constant permittivity ϵ_Si_ = 11.7 in the mid-IR (MIR) region was adopted, topped by the semi-infinite air layer.

A schematic representation of the top-view samples used for the measurements and the detection method is shown in Figure 3a. The measured second-order optical amplitudes of the O2A spectra for the three AlGaN/GaN samples and the GaN sample are compared to the modeled O2A in Figure 3b,c. The measured GaN spectrum was obtained from the AlGaN/GaN cross section by exclusively scanning the GaN part, while the simulated GaN is the modeled semi-infinite GaN. Also, the dips in the measured AlGaN/GaN spectra at around 730 cm^–1^, which corresponds to the GaN LO phonon resonance, agree considerably with the simulated spectra for all three samples investigated, showing a similar blue shift as the AlGaN thickness increases. A similar blue shift was reported in SrTiO3 nano-FTIR measured amplitude spectra and attributed to a change in the carrier concentration of the 2DEG.^46^ However, our simulated O2A spectra without implementing a 2DEG layer still show a blue shift at increasing AlGaN thickness (Figure S3). Thus, the effect observed on the O2A spectra cannot be fully attributed to the influence of the carrier concentration variation in the 2DEG and suggests that the interaction with the surrounding layers has a more prominent role for the O2A.

**Figure 3:**
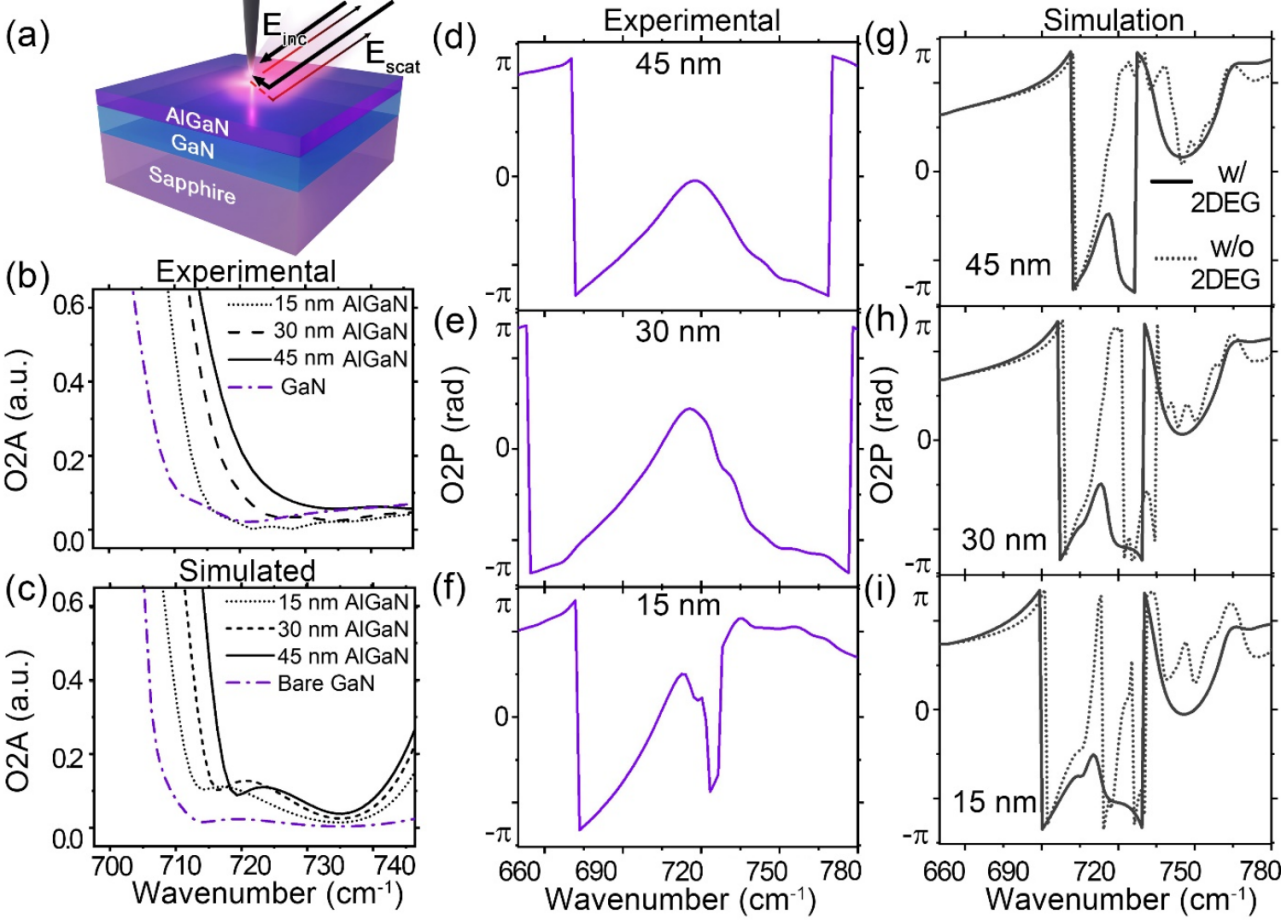
(a) Schematic example of the top-view sample along with the detection methods summarized as an AFM tip above the sample, the incident (*E*_inc_), and scattered (*E*_scat_) electric fields. (b) Simulated and (c) measured nano-FTIR O2A for the AlGaN/GaN samples with different AlGaN thicknesses. Bare GaN in the simulated data is the optical response of a semi-infinite GaN. (d–f) Measured and (g–i) simulated O2P spectra for the 15, 30, and 45 nm AlGaN samples.

When analyzing the O2P spectra, significant deviations from the measured spectra are observed when the 2DEG layers were not implemented in the simulated model, as shown in Figure 3d–i. The measured spectra (Figure 3d–f) are characterized by soft features between 690 and 720 cm^–1^ or wider. In contrast, the simulated spectra without the implementation of the 2DEG layers (dashed line in Figure 3g–i) have sharper and fast-varying features. Simulated spectra can reproduce similar features only when the 2DEG layers are included in the model. In fact, the measured and modeled phases show a relatively better agreement when the AlGaN thickness is 30 and 45 nm compared to when the thickness is 15 nm, reflecting that for thicker AlGaN layers, the effect of the increased carrier concentration in the simulated 2DEG layer is paramount for the correct interpretation of the measurements.

In essence, the free electrons at the 2DEG interface act partly as a mirror, reflecting the near-field, thus screening the scattering amplitude signal of the underlying GaN phonons while enhancing the peak at 760 cm^–1^ of AlN at the overlying AlGaN layer. Figure S3 shows the simulated O2A spectra without considering a virtual 2DEG layer; here, we can see how the peak at 760 cm^–1^ increases in intensity with increasing AlGaN thickness. Additional simulations where the AlN composition was increased to unity showed an increase of the same peak, providing additional evidence supporting the assignment of the peak to AlN. Previous studies of 2DEG heterostructures via nano-FTIR considered solely the amplitude spectra while disregarding the information on the phase spectra. The zero-crossing of the real part of the dielectric function is particularly sensitive to changes in the optical properties of the system, making it a powerful tool for investigating the properties of 2DEG. In our case, this region is located around the 720 cm^–1^ mark, where the phase spectra are particularly sensitive to the presence (or absence) of 2DEG, explaining why our study focuses on that region.

However, certain discrepancies in both amplitude and phase are observed when the measured and simulated spectra are compared, highlighting the limitations of the proposed approach. For instance, in the O2A spectrum, the AlN peak at 760 cm^–1^ predicted by the model is absent in the experimental data, where a corresponding feature appears blue-shifted to 800 cm^–1^ with reduced intensity, as shown in Figure S1a. Additionally, the simulated O2P spectrum exhibits a compression in frequency, whereas the experimental results display the same trend but over a significantly broader range. These discrepancies can be mitigated by adjusting the AlN longitudinal optical (LO) phonon frequency from approximately 880 to 700 cm^–1^, while keeping all other parameters of our model the same, hinting that the FDM model can widen the calculated phase by modifying the parameters already in the model, but as no valid physical reason has been found to adapt such an adjustment, we kept the utilized optical parameters as from the cited literature for all of the results shown in this work.

Apart from single-point measurements from the top, hyperspectral imaging was conducted on the cross section of the 45 nm AlGaN sample (Figure 4a), where the SEM image of the cross section is shown in Figure S4. The integrated amplitudes of O2A and O2P in the range of 700 and 737 cm^–1^ are shown in Figure 4b,c, respectively. The amplitude and phase decrease at around 75 nm in the *y*-axis, where the positions match the interface of AlGaN and GaN layers. The results clearly show that both amplitude and phase images can resolve the 2DEG layer. Together with the measured hyperspectral imaging, an FDM-based hyperspectral simulation was performed, and the integrated O2A and O2P images are shown in Figure 4d,e, respectively. The simulations show a similar trend to the experimental data when the modeled 2DEG layer thickness is 5 nm or above. If the 2DEG thickness is set to 2 nm, which is the typical 2DEG thickness for the AlGaN/GaN,^47^ the simulated O2P image shows a similar position dependence; however, the simulated O2A image has a different position dependence compared to the measured image (see Figure S5a,b). Thus, to reproduce the measured image, setting the 2DEG thickness to 5 nm or higher is necessary. Similarly, for AlGaN/GaN heterostructures, the 2DEG thickness has been arbitrarily adjusted to even 10 nm after considering electron density and wave function.^48–50^ The necessity to add a thicker thickness may come from a larger tip radius than the 2DEG thickness, and the reason behind this is that an increased tip radius reduces the spatial resolution, leading to a convolution of the signals due to the presence of the 2DEG and either or both of the neighboring layers (GaN or AlGaN). This change in the 2DEG layer thickness, however, does not affect the top-view spectra significantly, as shown in Figure S6.

**Figure 4:**
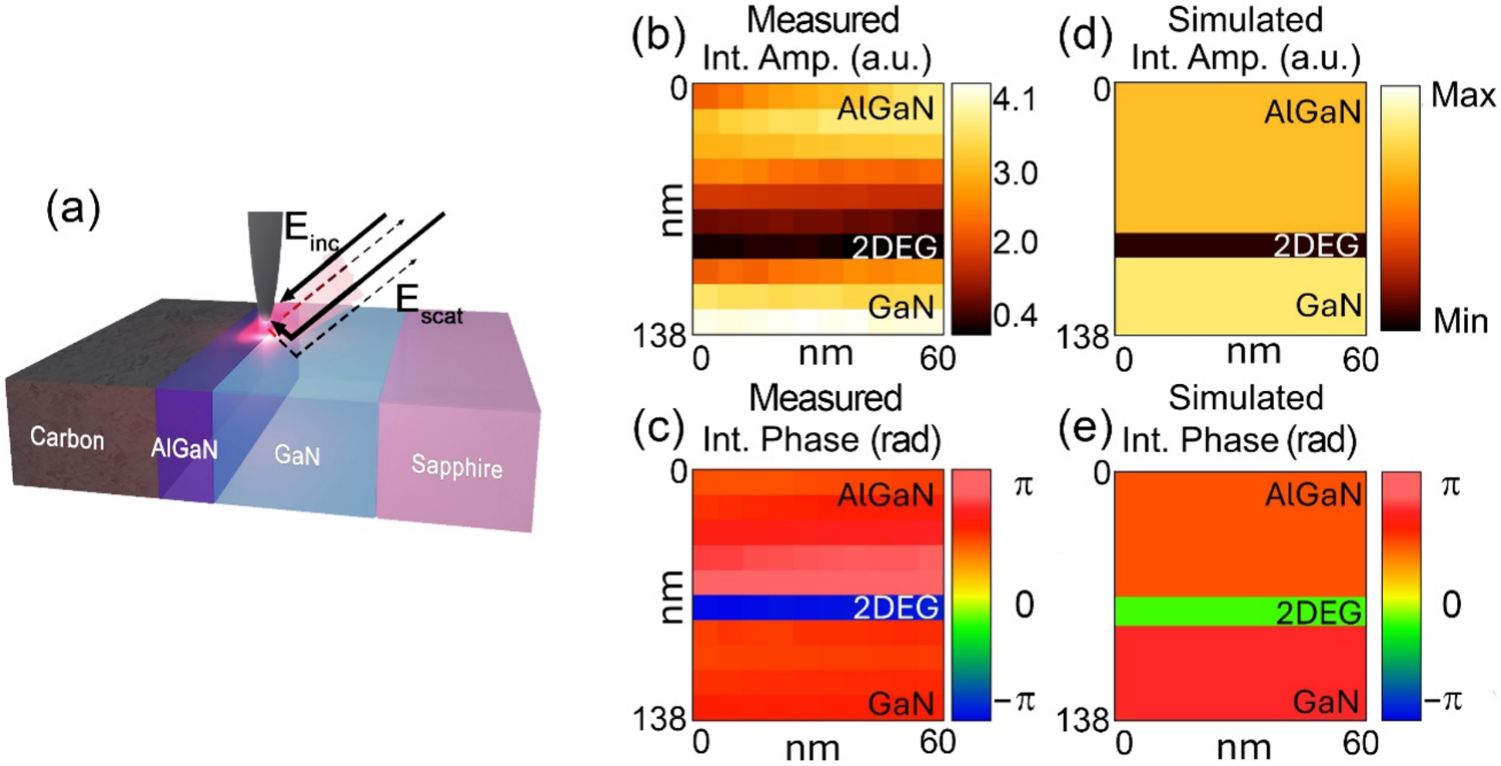
(a) Schematic of the cross-sectional sample with a representation of the detection method. Hyperspectral imaging of the integrated (b) O2A and (c) O2P of the 45 nm thick AlGaN sample for the range between 700 and 737 cm^–1^. Simulated hyperspectral images of (d) and (e) O2P.

In the following, far-field optical measurements, including reflectance and ellipsometry, are presented, which explored the possibility of detecting traces of the 2DEG layer. The reflectance of the three samples by the FTIR measurement and numerical simulations based on the finite element method are shown, respectively, in Figure 5a–c. The similarity between the measured and simulated spectra validates the measured results. They all possess similar features; the reflectance is not significantly different between the samples, and a negligible change is observed when adding the 2DEG layer in the simulation. Further validation comes from the measured ellipsometry data (Ψ and Δ) shown in Figure S7. The measured values of Ψ and Δ among the three samples show no significant variation. Hence, obtaining notable features on the 2DEG from the far-field measurements is impossible. This is caused by the fact that the incident MIR light at the far-field measurements propagated much deeper than the 2DEG layer, such that the reflected light was not sensitive to the existence of the 2DEG. In contrast, the limited probing depth of nano-FTIR allows the detection of the 2DEG, highlighting the advantage of near-field measurement.

**Figure 5:**
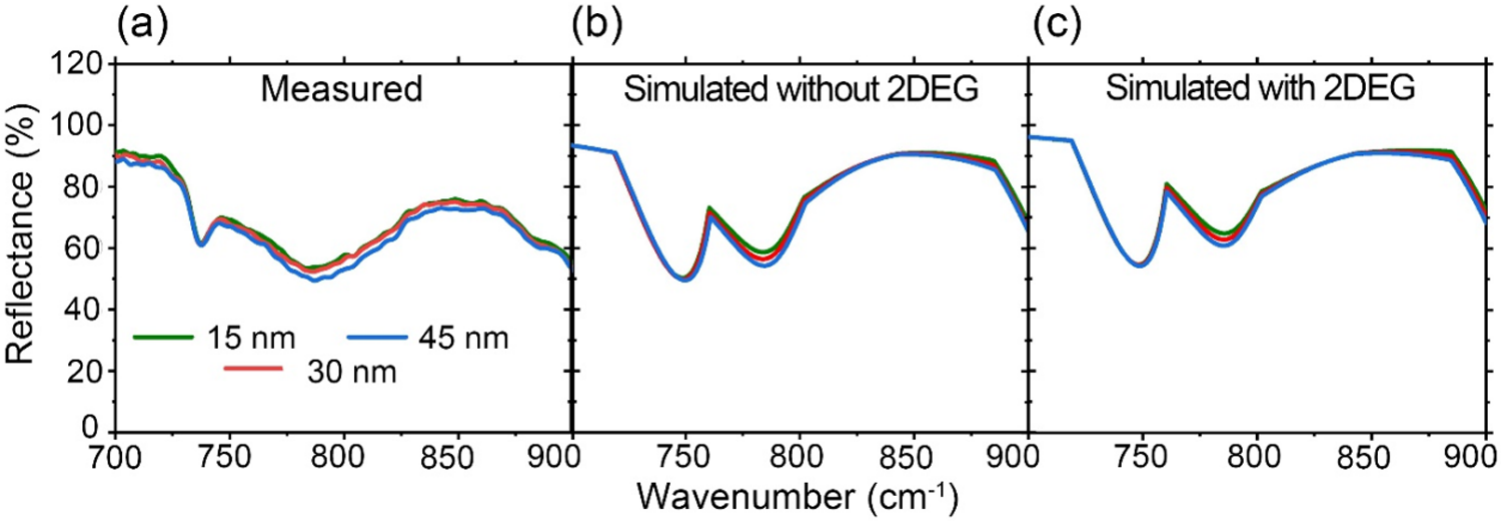
(a) Measured and (b, c) simulated reflectance of the AlGaN/GaN top-view samples with different AlGaN thicknesses. The simulations were conducted (b) without and (c) with the 2DEG layers.

## Conclusions

In summary, we successfully detected 2DEGs in AlGaN/GaN samples with AlGaN thicknesses from 15 to 45 nm through nano-FTIR spectroscopy. Although the major cause of the shifts in the O2A spectra was not due to the existence of 2DEG layers, these spectra clearly differentiate the AlGaN/GaN samples. Hyperspectral imaging directly visualized the spatial distribution and optical properties of the 2DEG layers, showing a stark contrast between the 2DEG layer and the surrounding GaN and AlGaN. This highlights the novelty of our work, as this is, to the best of our knowledge, the first nano-FTIR study on GaN/AlGaN 2DEGs that also includes cross-sectional sample analysis. The nanometer resolution and ambient operation offer a significant advantage over traditional 2DEG visualization methods, enhancing current characterization methodologies. We anticipate that nano-FTIR will be applied to characterize 2DEG layers in other samples, and further advances in analysis and modeling offer the potential to enhance the benefits of nano-FTIR.

## Supplementary Material



## Notes

Difference in simulation results between the charge average method and multilayer method (Figure S1); simulated approach curve, O2A and O2P spectra for different tip radii considered in the model (Figure S2); simulated O2A response without including the 2DEG layer in the model (Figure S3); SEM image of the AlGaN cross-sectional sample after Ar milling (Figure S4); simulated integrated O2A and O2P of the 45 nm thick AlGaN/GaN sample (Figure S5); differences of simulated spectra between 2 and 5 nm thick 2DEG layer (Figure S6); and measured ellipsometric data for the three AlGaN/GaN top-view samples (Figure S7) (PDF)
