# Supplementary material for: Infrared Near-Field Spectroscopy of AlGaN/GaN Heterostructures for Probing Two-Dimensional Electron Gas
Source: ACS Appl Mater Interfaces. 2025 Aug 22;17(35):50077–84. doi: 10.1021/acsami.5c12417 (PMC12412095; doi:10.1021/acsami.5c12417)
Supplement: Supplementary file 1 [file am5c12417_si_001.pdf]

Supporting Information

Infrared Near-Field Spectroscopy of AlGaN/GaN Heterostructures for Probing Two-Dimensional Electron Gas

Ilario Bisignano[1,2], Masataka Imura[3], Nicholaus Kevin Tanjaya[1,2], Ming-Jyun Ye[1,4], Noriyuki Okada[5], Satoshi Ishii[1,2,]*

1. International Center for Materials Nanoarchitectonics (MANA), National Institute for Materials Science (NIMS), Tsukuba, Ibaraki, 305-0044, Japan

2. Graduate School of Science and Technology, University of Tsukuba, Tsukuba, Ibaraki, 305-8577, Japan

3. Research Center for Functional Materials, National Institute for Material Science (NIMS), Tsukuba, Ibaraki, 305-0047, Japan

4. College of Photonics, National Yang Ming Chiao Tung University, Tainan, 711010, Taiwan

5. Research Network and Facility Services Division, National Institute for Materials Science (NIMS), Tsukuba, Ibaraki, 305-0047, Japan

*Corresponding Author

Email: sishii@nims.go.jp

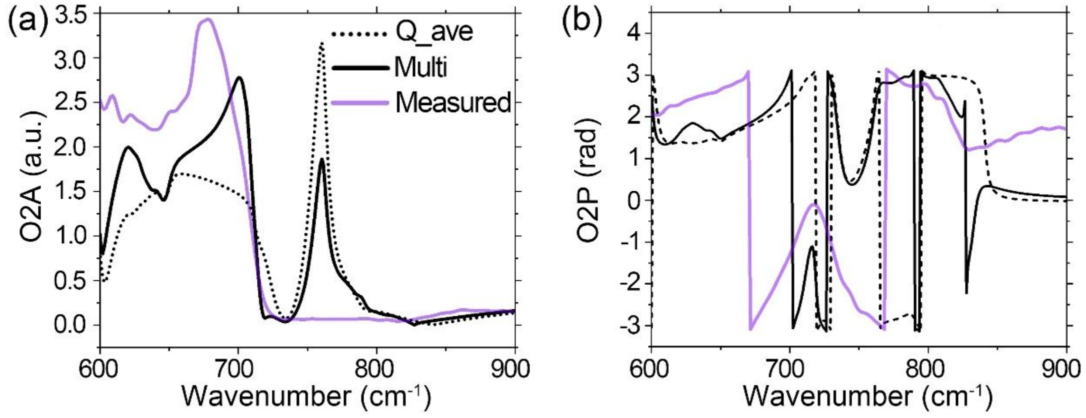

Figure S1. Comparison between the charge average method (Q_ave, in dotted lines) and multilayer method (Multi, in solid lines) for (a) O2A and (b) O2P calculated for the 45 nm thick AlGaN. The measured spectra are identical to the ones plotted in Figure 3 in the main text. In the multilayer method, preserving the normal electric field and potential gives the values of the effective depth and reflection coefficient. In the charge average method, the effective near-field reflection coefficient is derived by an effective point charge, reflected at the multilayered sample surface, and evaluated at the position of the point charge itself.

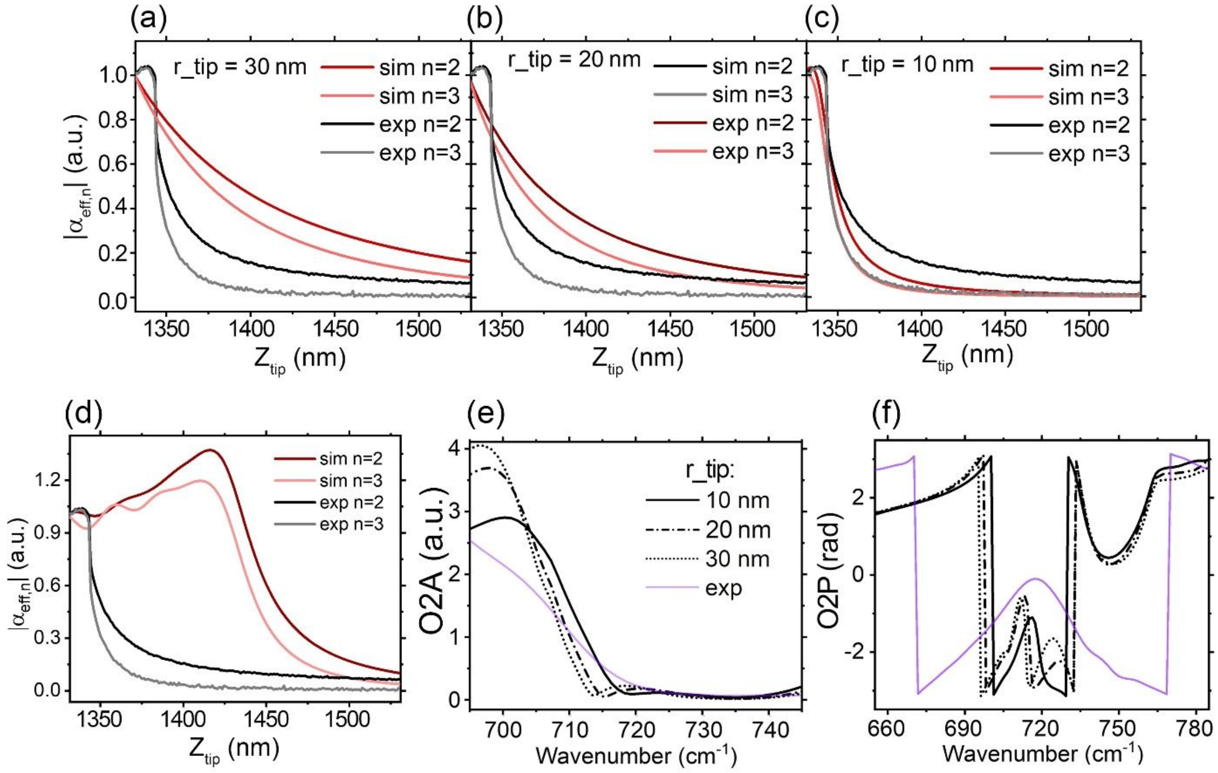

Figure S2. Approach curves of the simulated effective polarizability at second and third order harmonics when the simulated tip radii are (a) 30 nm, (b) 20 nm, (c) 10 nm and (d) 8 nm. The effective polarizability is defined as $\boldsymbol{\alpha_{eff}} \propto 1+\frac{\boldsymbol{f_0\beta_0}}{\boldsymbol{2(1-f_1\beta_0)}}$, where $\boldsymbol{\beta_j}$ is the quasistatic reflection coefficient of the j-th layer that depends on material permittivity and $\boldsymbol{f_j}$ accounts for geometrical parameters. The experimental approach curves are identical in each panel. (e-f) Simulated O2A and O2P for the 45 nm thick AlGaN sample with different tip radii, the 10 nm tip radius spectra overlap the best to the experimental data (violet solid line), further confirming the physical size of the probe.

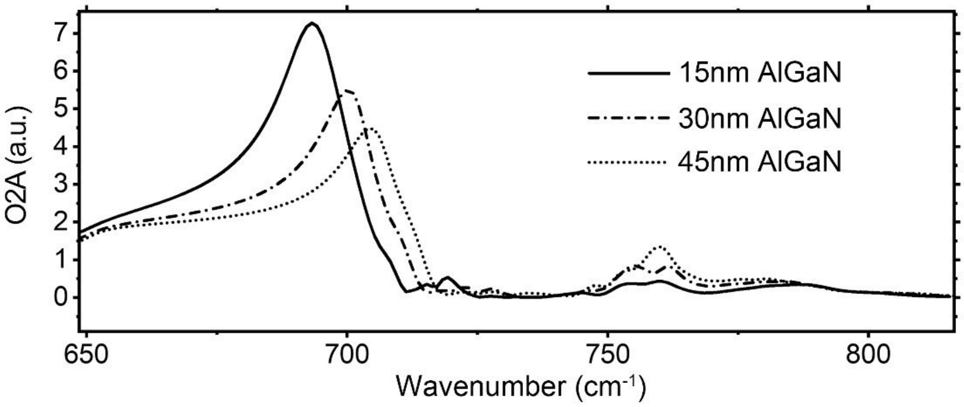

Figure S3. Simulated optical amplitude response for the three AlGaN/GaN top-view samples without the 2DEG layers.

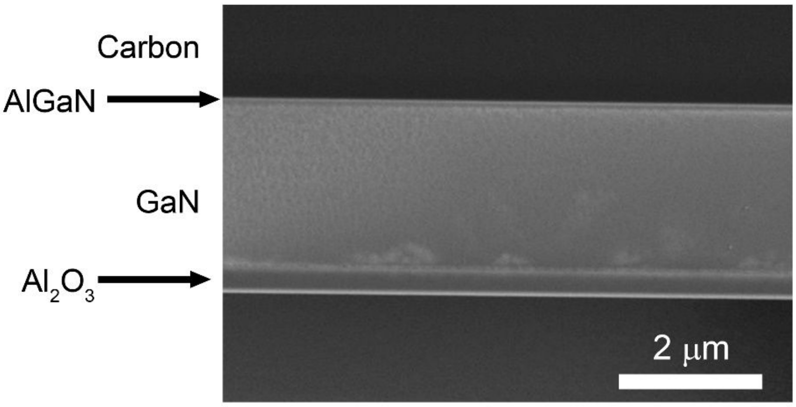

Figure S4. SEM image of the cross-section sample used for the hyperspectral imaging measurements after the Ar milling. Below the sapphire (Al_2O_3) is silicon, where the cross-section sample was loaded.

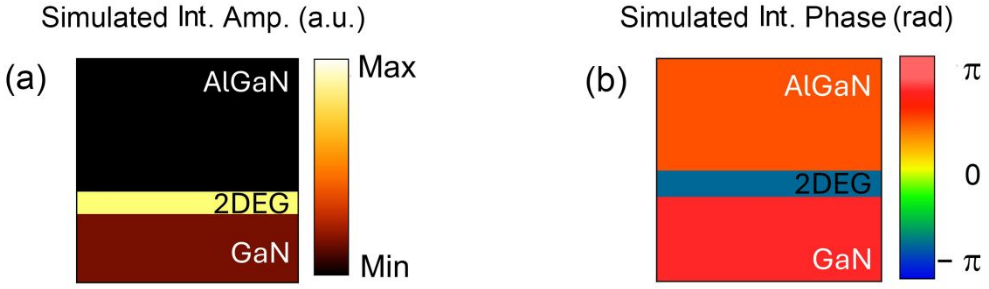

Figure S5. Simulated integrated second-order optical (a) amplitude and (b) phase of the 45 nm thick AlGaN/GaN sample with a 2 nm thick 2DEG layer. The O2A signal increases at the 2DEG layer, which is opposed to the measured image.

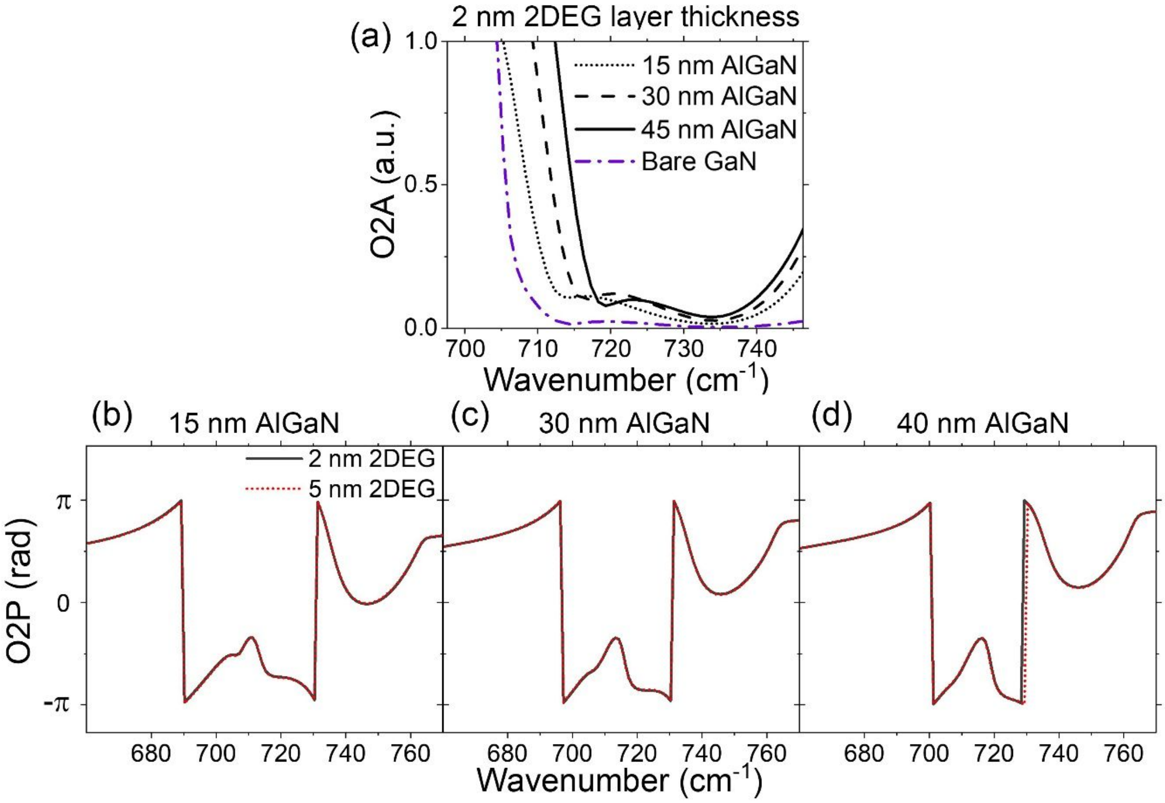

Figure S6. (a) Simulated O2A when the 2DEG layer thickness was set to 2 nm. Simulated O2P for (b) 15 nm AlGaN, (c) 30 nm AlGaN, (d) 45 nm AlGaN samples with 2DEG thicknesses of 2 nm and 5 nm. There is no notable difference between 2 nm and 5 nm thick 2DEG layers in the optical amplitude and phase spectra of the top-view samples within the considered spectral range.

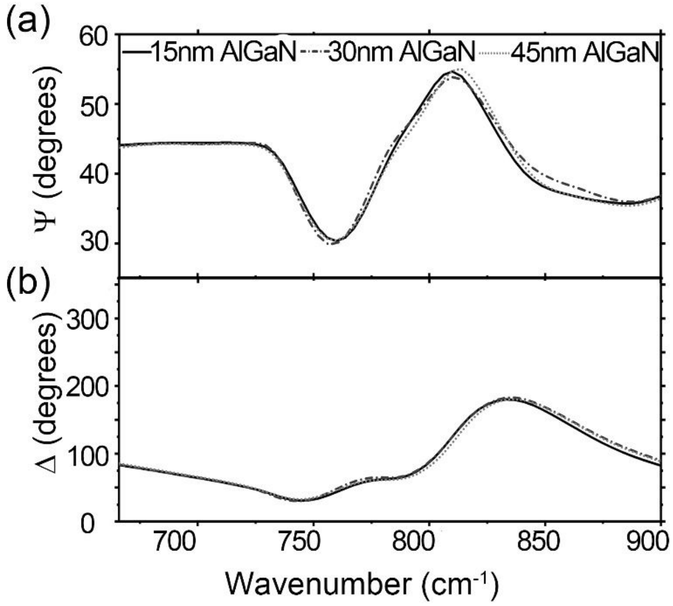

Figure S7. (a) ψ and (b) Δ obtained for the three AlGaN/GaN top-view samples measured with an infrared spectroscopic ellipsometer (SENDIRA, SENTECH Instruments GmbH).
